# Efficacy of Shear-Wave Elastography for Detecting Postoperative Cervical Lymph Node Metastasis in Papillary Thyroid Carcinoma

**DOI:** 10.1155/2018/9382649

**Published:** 2018-09-04

**Authors:** Hye Jeong Kim, In Ho Choi, So-Young Jin, Hyeong Kyu Park, Dong Won Byun, Kyoil Suh, Myung Hi Yoo

**Affiliations:** ^1^Division of Endocrinology and Metabolism, Department of Internal Medicine, Soonchunhyang University Hospital, Soonchunhyang University College of Medicine, Seoul, Republic of Korea; ^2^Department of Pathology, Soonchunhyang University Hospital, Soonchunhyang University College of Medicine, Seoul, Republic of Korea

## Abstract

**Aims:**

To evaluate shear-wave elastography (SWE) as a tool to detect postoperative cervical lymph node (LN) metastasis in patients with papillary thyroid carcinoma (PTC).

**Methods:**

Forty-three LNs of 43 patients with PTC undergoing ultrasound (US) and SWE before ultrasound-guided fine-needle aspiration (FNA) for the evaluation of postoperative cervical LN recurrences were analyzed. The mean (*E*_Mean_), minimum (*E*_Min_), maximum (*E*_Max_), and standard deviation (*E*_SD_) of SWE elasticity indices were measured.

**Results:**

Among 43 indeterminate or suspicious LNs, 12 were malignant and 31 were benign. The *E*_Mean_, *E*_Min_, *E*_Max_, and *E*_SD_ values were significantly higher in malignant LNs than in benign LNs (*E*_Mean_: 37.1 kPa in malignant versus 11.8 kPa in benign LNs, *P* < 0.001; *E*_Min_: 11.3 kPa versus 5.1 kPa, *P* = 0.046; *E*_Max_: 50.5 kPa versus 23.7 kPa, *P* < 0.001; and *E*_SD_: 7.8 kPa versus 4.1 kPa, *P* = 0.006). *E*_Max_ had the highest accuracy (93.0%) when applied with a cut-off value of 37.5 kPa. It had a positive likelihood ratio of 25.83 and a diagnostic odds ratio of 150.0.

**Conclusions:**

The shear elasticity index of *E*_Max_, with higher likelihood ratios for malignant LNs, may help identify postoperative cervical LN metastasis in PTC patients with indeterminate or suspicious LNs.

## 1. Introduction

Papillary thyroid carcinoma (PTC) accounts for approximately 80% of thyroid cancer. Postoperative metastasis usually occurs as cervical lymph node (LN) metastasis, and a regular checkup of cervical ultrasound (US) has been established as a routine procedure with serum thyroglobulin (Tg) measurement [1]. Therefore, the differentiation of malignant from benign LNs in PTC is very important to decide further management plans of the patients [1].

Conventional US is commonly used for diagnosing malignant cervical LNs. Usually, gray-scale US features including round shape of the LNs, microcalcification, loss of fatty hilum, hyperechogenicity, and cystic change suggest to be suspicious for malignant LNs, but variable portions (10~86%) of these LNs on US revealed thyroid cancer on fine-needle aspiration (FNA) or surgery [2]. So, there was no definite US criterion for diagnosing malignant LN metastasis with satisfactory sensitivity and specificity [3]. Recently, some studies have focused on providing the efficacy and diagnostic accuracy of shear-wave elastography (SWE) in the differential diagnosis of benign and malignant LNs but have reported on different SWE parameters with different cut-off values and variable inclusion of the study samples with different types of thyroid cancer [4–7].

The aim of our study was to investigate the role of elasticity indices as possible predictive parameters for postoperative cervical LN metastasis in PTC patients and to evaluate the diagnostic value of SWE for the differentiation of benign and malignant LNs in indeterminate or suspicious LNs on conventional US finding.

## 2. Methods

### 2.1. Study Population

From January 2016 to September 2017, a total of 237 patients with PTC who underwent US for postoperative evaluation were retrospectively reviewed for this study. Among these subjects, 48 were performed an US-guided FNA for indeterminate or suspicious LNs. Considering American [1], European [2], and Korean management guidelines for patients with thyroid nodules [8], we made the FNA criteria for LNs as follows: (1) a LN (≥5–7 mm in a short axis) having one of the findings for malignancy on US, such as cystic change, calcification, hyperechogenicity, or abnormal vascularity, and (2) a LN (≥8–10 mm in a short diameter) having indeterminate findings on US, such as loss of central hilar echo and absence of central hilar vascularity. For patients who had two or more LNs, a LN with suspicious sonographic appearance was aspirated preferentially. The US-guided FNA was performed for both cytological examination and Tg assay (IRMA kit; Cisbio Bioassay, Codolet, France: functional sensitivity 0.7 *μ*g/L) of the needle washout fluid. All patients underwent SWE prior to an US-guided FNA for LNs. Two experienced pathologists in the Department of Pathology confirmed the cytological and histological specimens. Metastatic LNs were defined as cytological specimen findings showing atypical cells or metastatic carcinomas and needle washout Tg > 10 ng/mL [9]. The diagnosis of metastatic LNs was confirmed by histological examination of the surgically resected specimens. The diagnosis of a benign LN was confirmed by the histological examination by FNA cytology and needle washout Tg results < 1 ng/mL [10]. Five LNs were excluded because they were characterized, based on the cytological specimen and needle washout Tg results, as having nondiagnostic/unsatisfactory (*n* = 1) or atypical/inconclusive result (*n* = 3) with needle washout Tg in an indeterminate range (1–10 ng/mL) or when patient refused the recommended surgical resection to have the final diagnosis (*n* = 1) with needle washout Tg > 10 ng/mL. Ultimately, the data from 43 LNs were included in this study.

### 2.2. Gray-Scale US and SWE Examinations

The patients were positioned for US with their necks extended. Each patient underwent gray-scale US and SWE using the Aixplorer US system (SuperSonic Imagine, Aix-en-Provence, France) and a linear probe with a frequency range of 4–15 MHz.

During gray-scale US examination, LNs were evaluated for size (width, depth, and length), volume, composition, echogenicity, shape, vascularity, and presence or absence of fatty hilum or calcification.

**Figure 1:**
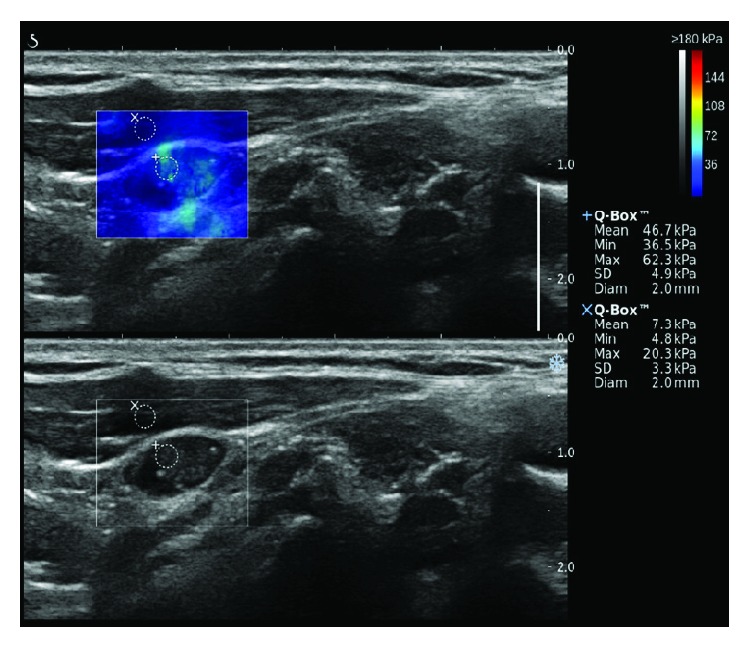
Shear-wave elastography (upper image) and gray-scale ultrasound (lower image) of metastatic cervical lymph nodes.

After the gray-scale US, SWE was performed by the same investigator who had also performed the gray-scale US, and the system was changed to the SWE to use the same probe to perform SWE. Gray-scale US and SWE images appeared simultaneously on two panels (Figure 1). The built-in region of interest (ROI) (Q-box; SuperSonic Imagine) of the system was set to include the LN and the surrounding tissue, which showed the semitransparent color map of the tissue stiffness overlaid on the gray-scale US image. The software generated and automatically calculated the mean (*E*_Mean_), minimum (*E*_Min_), maximum (*E*_Max_), and standard deviation (*E*_SD_) of the lesions' shear elasticity indices in kilopascals (kPa). A fixed 2 × 2 mm size of ROI was placed by an investigator over the visually stiffer region of the lymph node on the color map to obtain the *E*_Max_, selecting two or more fixed ROIs depending on the size or extent of the SWE color map. The representative values of the *E*_Mean_, *E*_Min_, *E*_Max_, and *E*_SD_ were obtained from the ROI with *E*_Max_ as the highest. The color map of the tissue stiffness ranged from dark blue (lowest stiffness; 0 kPa) to red (highest stiffness; 180 kPa).

### 2.3. Statistical Analysis

The LN size, elasticity values, and needle washout Tg of all the lesions are expressed as medians (25th and 75th percentile). Differences between benign and malignant LNs were compared using the Mann–Whitney *U* test. Group comparisons of categorical variables were performed using the *χ*^2^ test. For a small-cell value, Fisher's exact test was used. Results of categorical data are summarized using frequencies and percent values. We evaluated the sensitivity and specificity of the elasticity values to predict metastatic LNs using a receiver operating characteristic (ROC) curve analysis, estimating the area under the curve (AUC) with 95% confidence intervals (CI). The likelihood ratio demonstrates how many times more (or less) likely the patients with the disease have that particular result than patients without the disease [11, 12]. Likelihood ratios above 10 and below 0.1 represent strong evidence to rule in or rule out the diagnosis, respectively [11, 12]. All statistical analyses were performed using the SPSS Statistics 14.0 software package (Chicago, IL, USA). A *P* value of less than 0.05 was considered statistically significant.

### 2.4. Ethics Statement

The present study protocol was reviewed and approved by the Institutional Review Board of Soonchunhyang University Hospital (approval number 2016-05-002). The requirement for informed consent for this study was waived by the institutional review board because researchers only accessed the database for analysis purposes and because personal identifying information was not accessed.

## 3. Results

### 3.1. Comparison of Characteristics between Patients with Benign and Malignant Lymph Nodes in Papillary Thyroid Carcinoma

Of the 43 LNs, 12 (28%) were diagnosed as having malignant LNs and 31 (72%) as having benign LNs.

**Figure 2:**
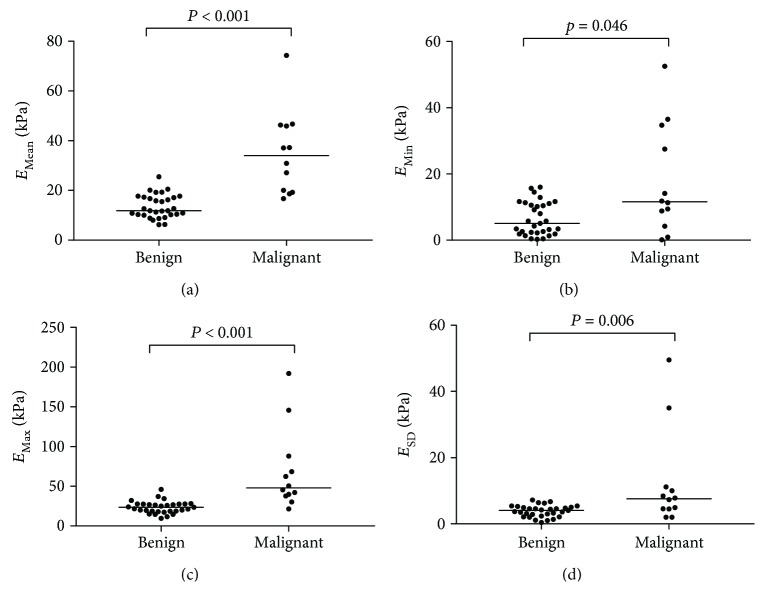
Scatter dot plots of shear-wave elastography measurements for benign (*n* = 31) and malignant (*n* = 12) lymph nodes in papillary thyroid carcinoma. (a) Mean elasticity (*E*_Mean_). (b) Minimum elasticity (*E*_Min_). (c) Maximum elasticity (*E*_Max_). (d) One standard deviation of elastographic values (*E*_SD_). kPa: kilopascal. Central bars denote median.

We compared the baseline characteristics of the patients with malignant LNs to those with benign LNs (Table 1). Gray-scale US features of calcification were significantly more frequently found in malignant LNs than in benign LNs (*P* = 0.002). Malignant LNs had higher elasticity values than benign LNs, which were statistically significant for *E*_Mean_ (37.1 versus 11.8 kPa, *P* < 0.001), *E*_Min_ (11.3 versus 5.1 kPa, *P* = 0.046), *E*_Max_ (50.5 versus 23.7 kPa, *P* < 0.001), and *E*_SD_ (7.8 versus 4.1 kPa, *P* = 0.006) (Figure 2). The median needle washout Tg levels were significantly higher in malignant LNs than in benign LNs (500.00 versus 0.08 ng/mL, *P* < 0.001). There were no significant differences between the groups with respect to gender, age at diagnosis, size, long/short diameter, hyperechogenicity, or presence of fatty hilum.

### 3.2. Diagnostic Value of Elasticity Indices for Predicting Metastatic Lymph Nodes in Papillary Thyroid Carcinoma

**Figure 3:**
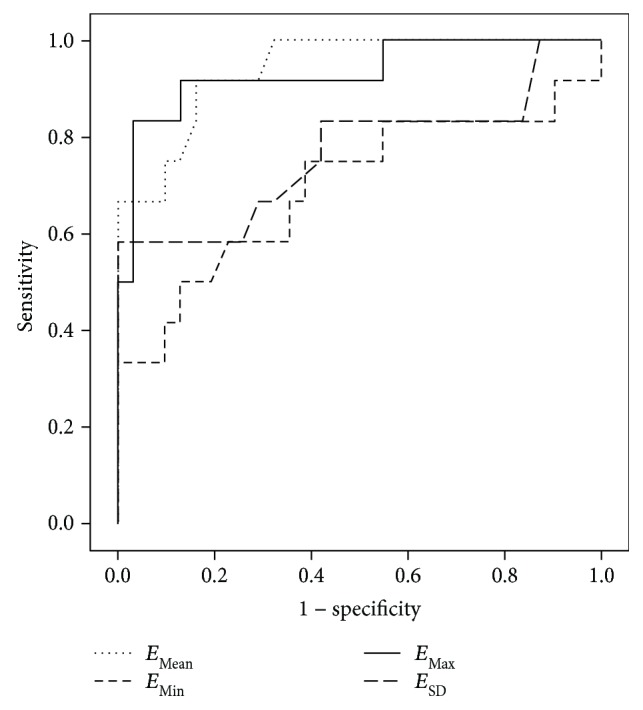
Receiver operating curves of *E*_Mean_, *E*_Min_, *E*_Max_, and *E*_SD_ for predicting malignant LNs.

The ROC analysis and diagnostic value of elasticity indices for discriminating benign and malignant LNs in PTC are presented in Table 2 and Figure 3. The *E*_Mean_, *E*_Min_, *E*_Max_, and *E*_SD_ values were significantly associated with malignant LNs and had diagnostic odds ratios ranging from 6.8 to 150.0. The sensitivity of elasticity indices associated with malignant LNs was somewhat low, ranging from 50.0% to 83.3%. However, the specificity of elasticity indices associated with malignant LNs was high, ranging from 87.1% to 96.8%. The positive predictive value ranged from 60.0% to 90.9%, and the negative predictive value from 81.8% to 93.8%. The positive likelihood ratio ranged from 3.87 to 25.83, and the negative likelihood ratio from 0.17 to 0.57. The *E*_Max_ showed the best diagnostic accuracy when applied at a cut-off value of 37.5 kPa, which had a specificity of 96.8%, a positive likelihood ratio of 25.83, and a diagnostic odds ratio of 150.0.

We further analyzed the diagnostic efficacy of the elasticity indices according to *E*_Max_ values (Table 3). A cut-off of *E*_Max_ > 21.4 kPa achieved 100% sensitivity but only 45.2% specificity. Conversely, a cut-off of *E*_Max_ > 48.4 kPa achieved 100% specificity but only 50.0% sensitivity.

## 4. Discussion

This study showed that the *E*_Mean_, *E*_Min_, and *E*_Max_ of SWE elasticity indices were significantly higher for malignant LNs than for benign LNs. The *E*_Max_ > 37.5 kPa analyzed alone had a clinically relevant positive likelihood ratio of 25.83 suggesting malignant LNs in PTC patients with indeterminate or suspicious LNs.

Several US features, such as cystic change, microcalcification, hyperechogenicity, or abnormal vascularity, have been associated with malignant LNs [2]. Diagnostic sensitivity ranges from 10% to 34% for cystic change, 5% to 69% for microcalcification, 30% to 87% for hyperechogenicity, and 40% to 86% for abnormal vascularity, whereas specificity ranges from 91% to 100%, 93% to 100%, 43% to 95%, and 57% to 93%, respectively [2]. There was no single US criterion for diagnosing malignant LNs with satisfactory sensitivity and specificity [3]. More recently, several studies evaluating the diagnostic values of SWE have suggested that the shear elasticity indices may be a useful tool for discriminating between benign and malignant LNs [4–7]. A pilot study by Bhatia et al. [4] demonstrated high diagnostic accuracy for malignant cervical LNs with an *E*_Max_ cut-off value of 30.2 kPa, with a sensitivity of 41.9% and a specificity of 100%. Choi et al. [5] reported that *E*_Max_ levels greater than 19.44 kPa provided a sensitivity of 91% and a specificity of 97%. Desmots et al. [7] also found, with excellent reproducibility, that the 31 kPa threshold in SWE was the best ultrasound sign to rule out malignant cervical LNs with a sensitivity of 87% and a specificity of 88%. However, these studies [4, 5, 7] included not only PTC but also various types of cancer, such as squamous cell carcinoma, nasopharyngeal cancer, and lymphoma. On the other hand, a study by Jung et al. [6] included only the LNs of preoperative or postoperative patients with PTC. They observed the highest specificity (100%) and PPV (100%) with *E*_Min_ (cut-off value of 24.0 kPa), the highest sensitivity (84.3%) with *E*_Max_ (cut-off value of 57.0 kPa), and the highest accuracy (72.6%) with *E*_Mean_ (cut-off value of 29.0 kPa). In the present study, we analyzed only indeterminate or suspicious LNs of postoperative patients with PTC to evaluate the usefulness of SWE for the cervical LN recurrences. The cut-off values of *E*_Mean_, *E*_Min_, *E*_Max_, and *E*_SD_ with optimal diagnostic performance for predicting malignant LNs were 23.0 kPa, 11.7 kPa, 37.5 kPa, and 6.9 kPa, respectively; these were lower than those reported by Jung et al. [6]. The present finding of lower cut-off values may be partly influenced by potential confounding factors of SWE such as LN size, calcifications, cystic changes, and the influence of precompression, position, or preoperative/postoperative approach.

In this study, the *E*_Max_ values showed the highest diagnostic accuracy and reproducibility among the elasticity indices. The *E*_Max_ of 37.5 kPa was the most accurate threshold, giving a relatively low sensitivity of 83.3% but a high specificity of 96.8%, a positive likelihood ratio of 25.83, and a diagnostic odds ratio of 150.0. The *E*_Max_ cut-off value of 37.5 kPa could provide strong evidence for the confirmation of a metastatic LN diagnosis (likelihood ratio above 10). On this basis, SWE may not be suitable for clinical practice for screening of LNs for malignancy. Conversely, high specificity can be useful in selecting LN subgroups that are more likely to be malignant and thus should undergo FNA regardless of conventional US findings.

The main limitation of our current study is its retrospective nature. There might have been selection bias because we only included indeterminate or suspicious cervical LNs that underwent US FNA. This is a single-center study with a limited number of patients. With the lack of multicenter evaluation, large prospective studies evaluating SWE parameters are anticipated to help verify our results, as well as the impact of SWE on the need for a LN FNA.

In conclusion, *E*_Max_, a shear elasticity index, had the highest diagnostic performance among all the SWE parameters for the detection of malignant cervical LNs in PTC patients. The SWE could be helpful for identifying postoperative cervical LN metastasis in PTC patients with indeterminate or suspicious LNs, as well as helpful for selecting LNs which require FNA in addition to the conventional US findings.

## Figures and Tables

**Table 1:** Comparison of baseline characteristics between patients with benign and malignant lymph nodes in papillary thyroid carcinoma (*N* = 43).

Characteristic	Benign (*n* = 31)	Malignant (*n* = 12)	*P* value
Female	26 (84%)	8 (67%)	0.405^∗^
Age (years)	51 (38, 64)	56 (35, 65)	0.606^†^
Size (cm)	1.2 (1.0, 1.6)	1.0 (0.8, 1.3)	0.152^†^
Long/short diameter	2.7 (1.8, 3.6)	2.2 (1.6, 2.6)	0.067^†^
Calcifications	3 (10%)	7 (58%)	0.002^∗^
Hyperechogenicity	5 (16%)	5 (42%)	0.087^∗^
Fatty hilum	10 (32%)	1 (8%)	0.107^∗^
Elasticity indices (kPa)			
*E*_Mean_	11.8 (10.3, 17.4)	37.1 (20.0, 46.3)	<0.001^†^
*E*_Min_	5.1 (2.0, 11.2)	11.3 (4.2, 34.7)	0.046^†^
*E*_Max_	23.7 (18.8, 27.6)	50.5 (39.9, 88.0)	<0.001^†^
*E*_SD_	4.1 (2.7, 5.0)	7.8 (4.6, 11.2)	0.006^†^
Needle washout Tg (ng/mL)	0.08 (0.02, 0.13)	500.00 (423.08, 4800.00)	<0.001^†^

kPa: kilopascal; *E*_Mean_: mean elasticity; *E*_Min_: minimum elasticity; *E*_Max_: maximum elasticity; *E*_SD_: one standard deviation of elastographic values; Tg: thyroglobulin. Data are presented as numbers (percentage) or medians (25th and 75th percentiles), as appropriate. Statistical significance was determined by the ^∗^*χ*^2^ test or ^†^the Mann–Whitney *U* test.

**Table 2:** ROC results and diagnostic performance of elasticity indices for predicting metastatic lymph nodes in papillary thyroid carcinoma.

Elasticity indices	Cut-off (kPa)	AUC	Sensitivity (%)	Specificity (%)	PPV (%)	NPV (%)	Accuracy (%)	PLR	NLR	Diagnostic odds ratio (95% CI)
*E*_Mean_	23.0	0.941	66.7	96.8	88.9	88.2	88.4	20.67	0.34	60.0 (5.9–614.2)
*E*_Min_	11.7	0.698	50.0	87.1	60.0	81.8	76.7	3.87	0.57	6.8 (1.4–31.6)
*E*_Max_	37.5	0.933	83.3	96.8	90.9	93.8	93.0	25.83	0.17	150.0 (12.3–1836.3)
*E*_SD_	6.9	0.769	58.3	96.8	87.5	85.7	86.0	18.08	0.43	42.0 (4.2–418.6)

kPa: kilopascal; AUC: area under the ROC curves; CI: confidence intervals; PPV: positive predictive value; NPV: negative predictive value; PLR: positive likelihood ratio; NLR: negative likelihood ratio; *E*_Mean_: mean elasticity index; *E*_Min_: minimum elasticity index; *E*_Max_: maximum elasticity index.

**Table 3:** Discrimination of metastasis from benign lymph nodes in papillary thyroid carcinoma using different cut-offs.

Elasticity indices	Cut-off (kPa)	Sensitivity (%) (95% CI)	Specificity (%) (95% CI)	PLR	NLR
*E*_Max_	21.4	100.0 (75.8, 100.0)	45.2 (29.2, 62.2)	1.82	0.00
	29.4	91.7 (64.6, 98.5)	87.1 (71.2, 94.9)	7.10	0.10
	37.5^a^	83.3 (55.2, 95.3)	96.8 (83.8, 99.4)	25.83	0.17
	48.4	50.0 (25.4, 74.6)	100.0 (89.0, 100.0)	—	0.50

kPa: kilopascal; CI: confidence intervals; PLR: positive likelihood ratio; NLR: negative likelihood ratio; *E*_Max_: maximum elasticity index. ^a^Threshold with the highest accuracy.

## Data Availability

The data used to support the findings of this study are available from the corresponding author upon request.
